# Is Genetic Differentiation Involved in the Morphological Adaptation of *Adenophora triphylla* var. *japonica* (Camanulaceae) to Water Flow Stress Along Rivers?

**DOI:** 10.1002/ece3.72222

**Published:** 2025-09-28

**Authors:** Iori Yajima, Masayuki Shiba, Kyohei Ohga, Yoshimasa Kumekawa, Tatsuya Fukuda

**Affiliations:** ^1^ Graduate School of Integrative Science and Engineering Tokyo City University Tokyo Japan; ^2^ Miyoshi City Hall Tokushima Japan; ^3^ The United Graduate School of Agricultural Sciences Ehime University Nankoku Kochi Japan

**Keywords:** *Adenophora triphylla*, genetic differentiation, morphological adaptation, phenotypic plasticity, rheophyte, riverside habitat, water flow stress

## Abstract

Plants along rivers have narrow lanceolate leaves, flexible stems, and petioles to avoid the water flow stress caused by flooding. This study aimed to determine whether the adaptation of *Adenophora triphylla* (Thunb.) A.DC. var. *japonica* (Regel) H. hara (Campanulaceae) with narrow leaves in riverside habitats was achieved through phenotypic plasticity or genetic morphological changes, where we conducted comparative morphological analyses through cultivation experiments. Our cultivation experiments revealed that the morphology of radical leaves in the riverside population had significantly smaller and narrower laminas and shorter and thicker petioles than those in the inland population, and that the former had significantly more radical leaves than the latter, suggesting that the former brings the total leaf area closer to that of the latter by increasing the number of radical leaves. The cauline leaves were significantly thinner and smaller in the riverside population than in the inland population, and the stems of the former were significantly shorter and thicker than those of the latter. In addition, a significant difference was observed between the riverside and inland populations in the number of rosette leaf branches from the rhizome, with the former having significantly more rosette leaf branches. Our results reveal that populations of *Ad*. *triphylla* var. *japonica* with genetically distinct leaf and stem morphologies have become established along rivers, where flooded water imposes strong selective pressure. In these riverside populations, thicker and shorter petioles and stems appear to reduce bending moments without breaking, while narrower and smaller laminas of both radical and cauline leaves further contribute to this reduction.

## Graphical abstract

This study found that riverside populations of Adenophora triphylla have genetically fixed morphological traits—such as narrower leaves and thicker, shorter petioles and stems—that help resist water flow stress. These traits were maintained even under cultivation, suggesting adaptive evolution rather than mere plasticity.

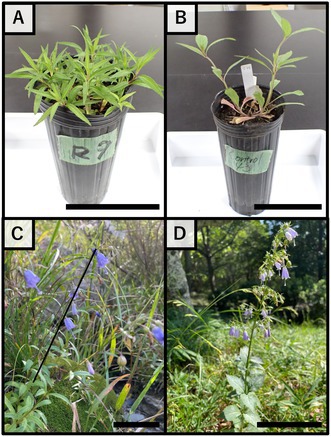

## 1 Introduction

Various plant traits are observable characteristics that reflect adaptive responses to environmental conditions (Poorter et al. 2009). In particular, the morphological characteristics of leaves have often been used for species identification for several years (Foster and Gifford 1989; Cope et al. 2012), but they also change depending on the environment (Givnish and Vermeij 1976; Royer and Wilf 2006; Nicotra et al. 2011; Schmerler et al. 2012). Leaf traits reflect the growing environment (Johnson 1975; Ellenberg 1985; Yeats and Rose 2013; Goldsmith et al. 2017; Grace 2019), and recent studies have documented variations in the morphology and anatomy of leaf traits along environmental gradients (Hayakawa et al. 2012; Tunala et al. 2012; Ohga, Muroi, Hayakawa, Yokoyama, et al. 2012; Ohga et al. 2013; Sunami et al. 2012; Kumekawa et al. 2013; Shiba et al. 2022a, 2022b, 2022c; Takizawa et al. 2022). It is, therefore, necessary to examine whether these morphologies change along the environmental gradients.

Understanding the ability of plants to adapt to diverse environments is at the core of ecological research. Natural populations can avoid extinction through phenotypic plasticity and adaptive evolution in the face of environmental change. Phenotypic plasticity can lead to large changes in the appearance or metabolism of an individual, such as those occurring in response to salinity and water stress (Winter and Ziegler 1992; Nosek et al. 2021; Takizawa et al. 2023), and is often sufficient to influence fitness (Bonser 2021). For example, several studies have reported that plants can respond to volatile organic compounds released by herbivore‐damaged plants to adjust their defenses before they invade, or use them to warn neighboring plants of impending danger (Heil and Karban 2010; Arimura 2021). Such biotic stresses involve metabolic changes in plants, whereas abiotic stresses often affect plant morphology. Among the various abiotic stressors, wind is one of the most ubiquitous, and it has been reported that different wind speeds in various environments affect plant morphology (Gardiner et al. 2016). Wind acts on plants above ground and causes stress, but plant responses are manifested directly above ground and indirectly below ground; for example, wind has been found to change shoot morphological traits, such as plant height (Nagashima and Hikosaka 2011; Feng et al. 2019) and leaf morphology (Anten et al. 2010; Shiba et al. 2023) above ground, as well as root morphological traits below ground (Werger et al. 2020). These studies suggest that wind intensity affects all growth forms, including trees, herbs, and weeds, at all life stages, and much is known about its natural and ongoing effects on plant morphology (de Langre 2008; Gardiner et al. 2016). Furthermore, Shiba, Kobayashi, et al. (2024) showed that phenotypic plasticity leads to differences in seed number through adaptive changes in leaves, stems, and roots to avoid or resist wind. Plants often adapt to wind stress by altering their organs through phenotypic plasticity (Cleugh et al. 1998; Poorter et al. 2012; Gardiner et al. 2016; Feng et al. 2019; Shiba et al. 2023; Shiba, Kobayashi, et al. 2024). Plants also adapt to various other abiotic stresses through phenotypic plasticity.

The abiotic stresses along the river vary in frequency and size, and plants in these areas have been largely affected by flooding from heavy rains, wind storms, and typhoons (Sakio et al. 2008). Therefore, they have a characteristic morphology, such as narrow lanceolate leaves, matted root systems, and flexible stems and petioles, to avoid flash floods (van Steenis 1981; Imaichi and Kato 1997; Sharpe 1997). Plants with narrow lanceolate leaves that grow along rivers often have specialized habitats and appear in parallel with various plant groups. This type of ecological group is called a rheophyte (van Steenis 1981). A comparative study of *Osmunda lancea* Thunb. (Osmundaceae) and its closely related inland species ( *O. japonica* ) indicated that there is a strong correlation between gross morphology and the anatomy of pinnules, and that stenophyllization in this species appeared to be caused by a decrease in cell size in the leaf‐width direction (Imaichi and Kato 1992). More recently, Shiba and Fukuda (2024) revealed that the petioles of *O. lancea* are flexible. Such research has been conducted not only on ferns but also on angiosperms; for example, Usukura et al. (1994) concluded that the narrow leaves of *Farfugium japonicum* (L.) Kitam. var. *luchuense* (Masam.) Kitam. (Asteraceae) evolved because of a decreased number of leaf cells across the width of the leaf. Moreover, *Rhododendron indicum* (L.) Sweet f. *otakumi* T. Yamaz. and *R. ripense* Makino (Ericaceae) are only involved in decreasing the number of leaf cells (Setoguchi and Kajimura 2004; Ueda et al. 2012), and the narrow leaves of *Viola mandshurica* W. Becker var. *ikedaeana* (W. Becker, ex Taken.) F. Maek. (Violaceae) are also caused by a decreased number of cells (Matsui et al. 2013). Although the narrow lanceolate or cuneate leaves are formed by changes in the number of leaf cells, Tsukaya (2002) and Yamada et al. (2011) reported variations in leaf width in *Dendranthema yoshinaganthum* (Makino ex Kitam.) Kitam. and *Aster microcephalus* (Miq.) Franch. et Sav. var. *ripensis* Makino that are involved in determining both the size and number of leaf cells. Several other studies have been conducted (Yokoyama et al. 2012; Shiba et al. 2021), and various morphological characteristics of plants growing along rivers have been studied. Although these adaptive morphologies enable growth along rivers in the face of rising and flooding river water, these characteristic traits allow them to adapt to abiotic stresses through phenotypic plasticity. In general, studies on the relationship between environmental stress and plant plasticity have identified unique plasticity mechanisms by comparing wild and cultivated plant species (Funk 2008; Nicotra and Davidson 2010; Brachi et al. 2012; Richter et al. 2012; Gratani 2014; Shiba, Kobayashi, et al. 2024). Thus, the extent of plasticity in wild plants under environmental stress can be compared with that in individuals obtained through cultivation under stress relief, allowing us to discuss the similarities and differences in plastic responses between the two groups with a focus on environmental stress. In addition, analysis of phenotypic plasticity through comparative studies using wild and cultivated populations may provide answers regarding the mode of adaptation to mechanical stress in plants growing in riverside environments.

**FIGURE 1:**
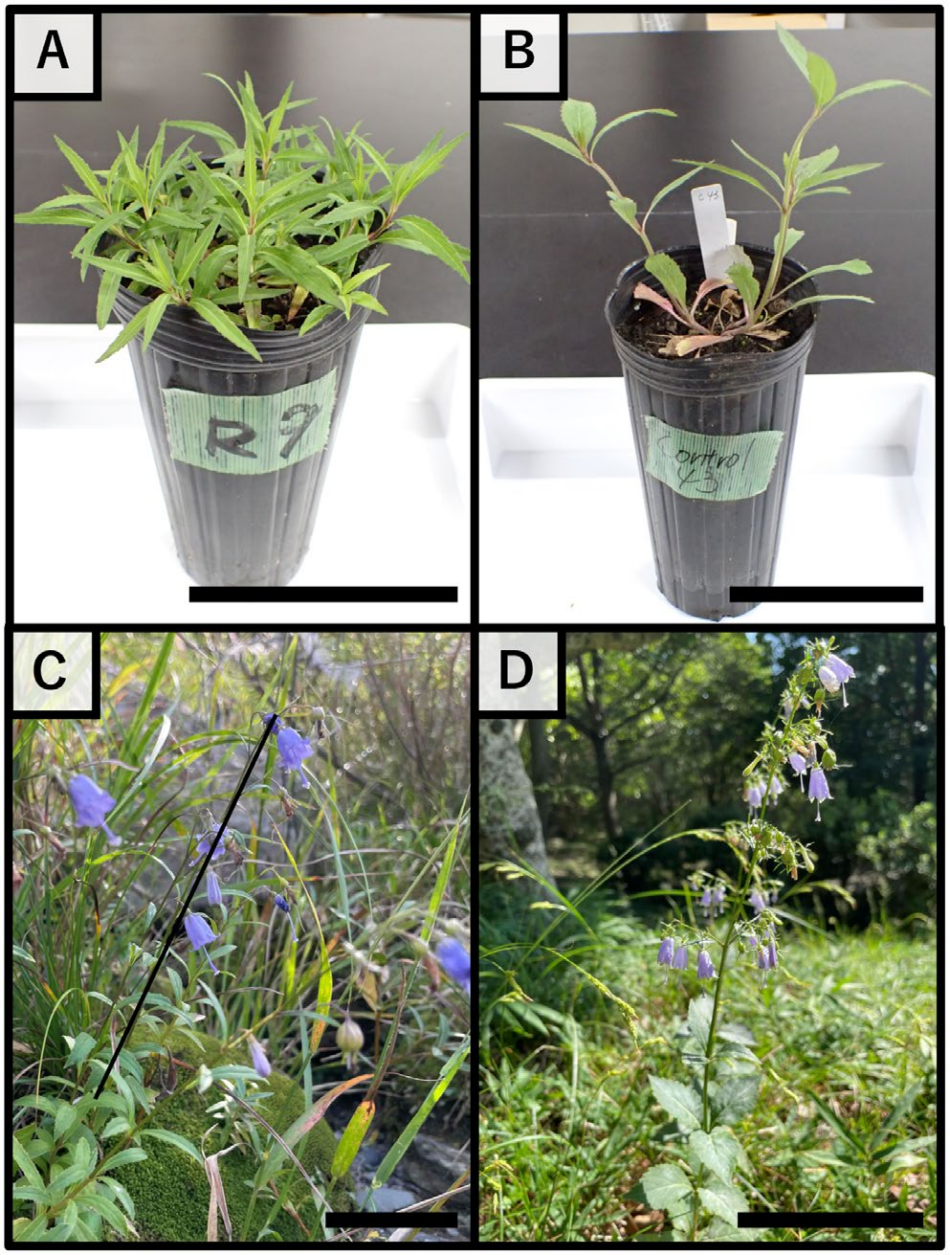
*Adenophora triphylla* var. *japonica*. Cultivation of (A) riverside and (B) inland. Wild of (C) riverside and (D) inland. Scale bar = 10 cm.

*Adenophora triphylla* (Thunb.) A.DC. (Campanulaceae) has been used as a traditional herbal medicine in East Asia to treat various illnesses, including cough, phlegm, asthma, and airway inflammatory diseases (Kim and Kim 2021), and its extracts contain diverse phytochemicals, such as saponin, inulin, polystycol, lupenone, β‐sitosterol, triphyllol, and daucosterol, which have been reported to exert various beneficial effects, such as anti‐obesity, antioxidant, anti‐fungal, anti‐melanogenic, anti‐inflammatory, and anti‐tumor activities (Lee et al. 2013; Kim and Kim 2021; Park and Park 2023). *Ad. triphylla* contains four subspecies, var. *triphylla*, var. *japonica* (Regel) H.Hara, var. *sasamotoi* Sugim, and var. *puellaris* (Honda) H. hara, and *Ad. triphylla* var. *japonica* is a perennial plant with almost unbranched stems 40–100 cm tall, which grows in grasslands in the mountains and fields of Japan, Korea, and Sahalin (Okazaki 1993) (Figure 1). This species forms radical leaves after sowing, but they die at the flowering stage, and from the following year, the stem leaves grow in whorls of 3–4 leaves, ovate‐elliptical, 4–8 cm long, with teeth on the leaf margins, and very short petioles (Okazaki 1993). The flowering season of this species is from August to October, and the racemes form at the apex of the stem. The pale purple to white, 1.5–2 cm long, bell‐shaped flowers grow in whorls slightly downwards, with fused sepals that are five‐lobed, and the sepal lobes are linear and 3–5 mm long (Okazaki 1993). Yamanaka and Takezaki (1959) reported that populations with narrow lanceolate leaves of *Ad. triphylla* var. *japonica* are found along several rivers in Japan. Ohga, Muroi, Hayakawa, Ito, et al. (2012) reported that the riverside population of *Ad. triphylla* var. *japonica* adapts to riverside environments as a rheophytic ecotype by forming narrow lanceolate leaves and decreasing both the size and number of leaf cells. The question then arises as to whether the narrow lanceolate leaves of the riverside populations of *Ad. triphylla* var. *japonica* are phenotypic plasticity, influenced by water flow stress during leaf growth, or whether this group has genetically narrow lanceolate leaves. Therefore, this study aimed to clarify the adaptive morphological characteristics of *Ad. triphylla* var. *japonica* associated with riverside adaptation, using wild population data from Ohga, Muroi, Hayakawa, Ito, et al. (2012) and cultivated populations from this study.

## 2 Materials and Methods

### 2.1 Seed Collection and Cultivation Conditions

The seeds of the rheophytic ecotype of *Adenophora triphylla* var. *japonica* used in this study were collected from a population along the Yoshino River in Motoyama, Motoyama‐cho, Nagaoka‐gun, Kochi Prefecture (33°76′N, 133°60′ E), the same site as the population used by Ohga, Muroi, Hayakawa, Ito, et al. (2012). The seeds of the inland population were collected from a population in Shitōridoi, Minamiawaji City, Hyogo Prefecture (34°20′N, 134°48′ E).

On April 6, 2023, a cultivation experiment was conducted at Tokyo City University. Seeds from both groups were sown in vertically oriented pots with a capacity of 1,600 mL filled with black soil. The study was conducted until the second year, when stem and cauline leaf formation occurred. The total number of samples used in this study was 35 individuals from the riverside population and 92 individuals from the inland population.

### 2.2 Morphological Measurements

Morphological measurements were conducted in September 2024. Radical and cauline leaves were photographed using a digital camera (Tough TG‐6; OM Digital Solutions Corporation, Tokyo, Japan). Lamina length (mm), lamina width (mm), angle at lamina base (°), and lamina area (mm^2^) were analyzed using the image analysis software ImageJ. The leaf index was calculated as the ratio of lamina length to lamina width. Petiole length (mm) was measured three times using a 15 cm ruler (model 14,001; Shinwa Rules Co. Ltd., Niigata, Japan), and the average value was calculated. The basal diameter of the petiole (mm) was measured three times using a digital caliper (CD‐15APX; Mitutoyo Corporation, Kanagawa, Japan), and the average value was calculated. The number of leaves per individual was also counted. Stem length (cm) was measured using a 60 cm ruler (model 14,036; Shinwa Rules Co. Ltd., Niigata, Japan). The basal stem diameter (mm) was measured using a digital caliper (CD‐15APX; Mitutoyo Corporation, Kanagawa, Japan). In addition, the number of rosette‐leaf branches per individual was also counted.

To determine whether the morphological phenotypes of cauline leaves and stems are genetically fixed, a comparison was made with the values from the field populations of Ohga, Muroi, Hayakawa, et al. (2012).

### 2.3 Statistical Analysis

Statistical comparisons between two samples were conducted using Microsoft Excel. An F‐test was first used to assess the homogeneity of variances between the two samples. Based on the results of the *F*‐test, a *t*‐test was performed under the assumption of either equal or unequal variances. Relationships between two continuous variables were analyzed using the statistical software R. The coefficient of determination (*R* ^2^) and the regression equation were calculated, followed by regression analysis. To evaluate the applicability of ANCOVA between two groups, the normality of the data was tested.

## 3 Results

In the relationship between the two variables, a natural logarithmic transformation was applied to improve normality. After the transformation, normality improved compared to the original values.

### 3.1 Morphological Measurements of the Lamina and Petiole of the Radical Leaves

**FIGURE 2:**
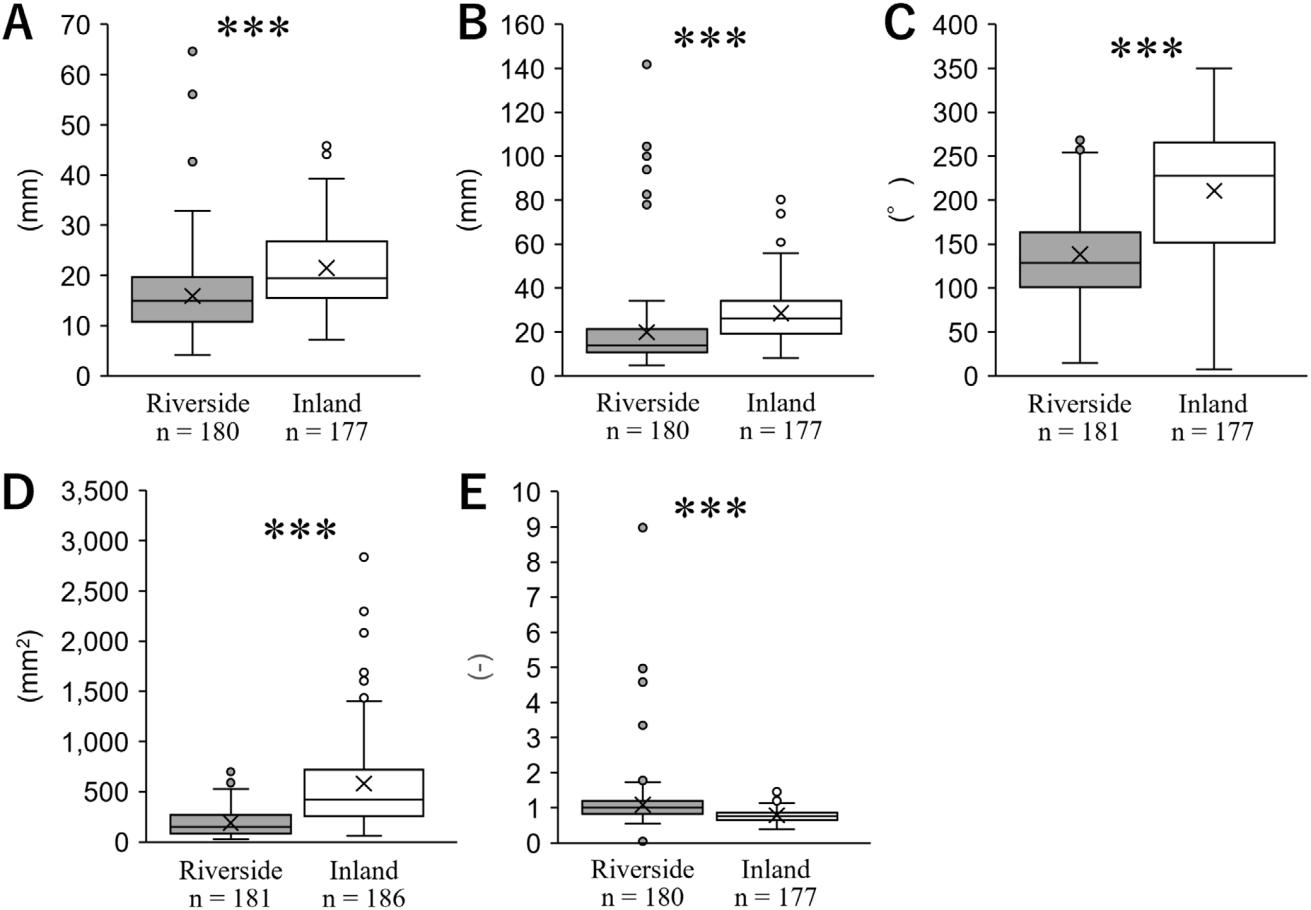
Comparisons of lamina of radical leaves in cultivation. (A) length, (B) width, (C) angle at lamina base, (D) leaf area, (E) leaf index. The statistical significance is as follows: ***, *p* < 0.001, n.s., not significant.

The leaf length of the riverside population was 15.89 ± 0.58 (mean ± SE), whereas that of the inland population was significantly longer at 21.47 ± 0.63 (*p* < 0.001; Figure 2A). The leaf width of the riverside population was 19.81 ± 1.50, while that of the inland population was significantly wider at 28.43 ± 0.92 (*p* < 0.001; Figure 2B). The angle at the lamina base of the riverside population was 138.27 ± 3.79, whereas the inland population had a significantly wider angle at 210.66 ± 5.58 (*p* < 0.001; Figure 2C). The leaf area of the riverside population was 188.31 ± 9.82, whereas that of the inland population was significantly larger at 581.75 ± 35.90 (*p* < 0.001; Figure 2D). The leaf index of the riverside population was 1.09 ± 0.06, while that of the inland population was significantly higher at 0.78 ± 0.01 (*p* < 0.001; Figure 2E). These results indicate that the lamina of the radical leaves in the riverside population has a smaller area and an elongated shape, which is genetically fixed (Figure 2).

**FIGURE 3:**
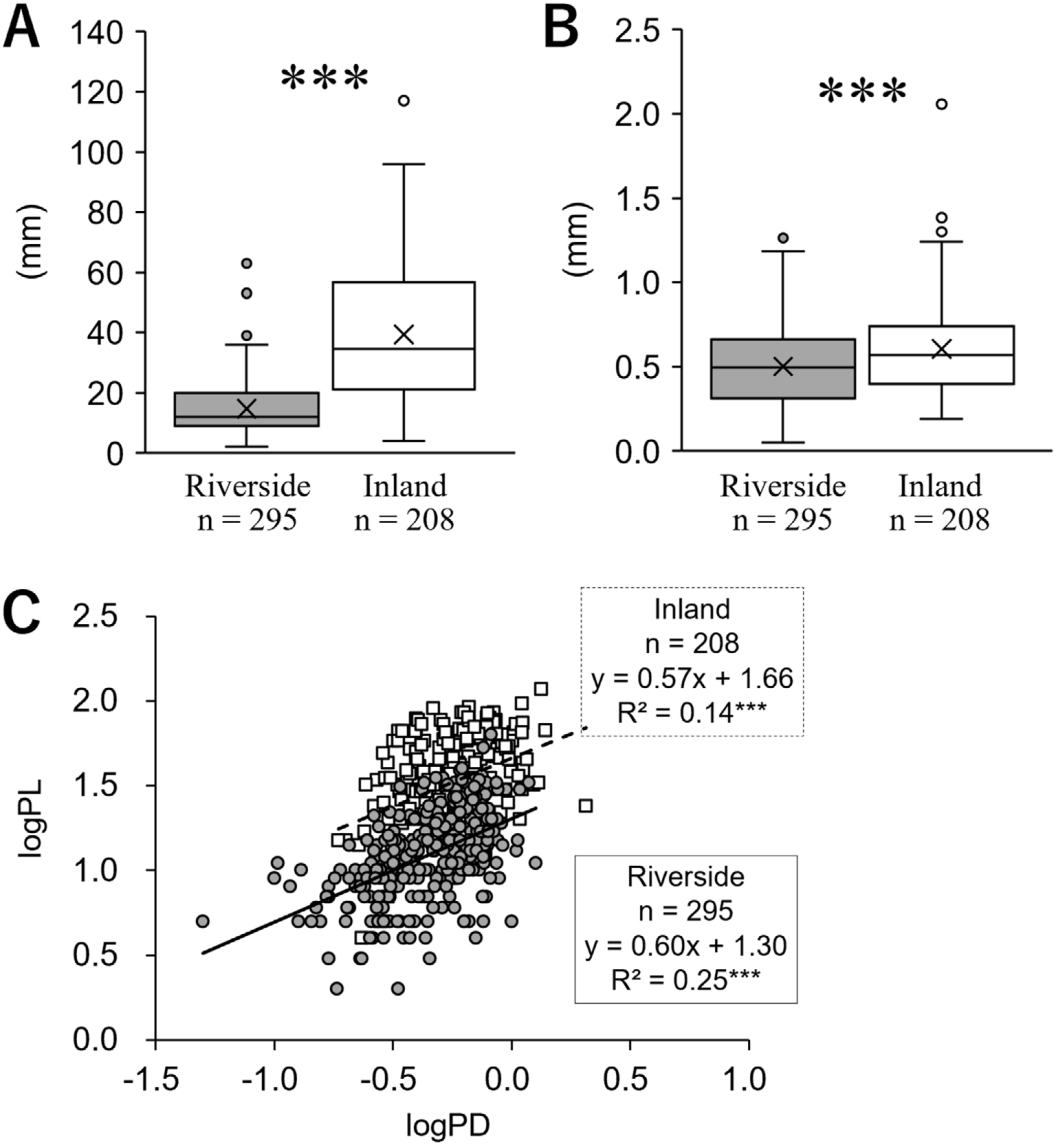
Comparisons of petioles of radical leaves in cultivation. (A) length, (B) basal diameter. (C) Correlation between basal diameter (logPD) and length (logPL) of radical leaves. The riverside population is indicated by grey circles and solid lines, while the inland population is indicated by white squares and dashed lines. The statistical significance of comparisons between two groups and the relationship between two variables is as follows: ***, *p* < 0.001, n.s., not significant.

The petiole length of the riverside population was 14.71 ± 0.51, whereas that of the inland population was significantly longer at 39.30 ± 1.54 (*p* < 0.001; Figure 3A). The diameter at the base of the petiole in the riverside population was 0.49 ± 0.01, while that of the inland population was significantly thicker at 0.60 ± 0.01 (*p* < 0.001; Figure 3B). The relationship between petiole diameter at the base and petiole length showed a statistically significant positive correlation in both populations (riverside: *p* < 0.001, inland: *p* < 0.001). Additionally, the results of ANCOVA revealed a significant difference in the intercept (*p* < 0.001), but no interaction effect was observed (*p* = 0.74). This suggests that the slope of the relationship between petiole length and diameter at the base is the same regardless of the growing environment, while the intercept of petiole length differs depending on the environment (Figure 3C). Furthermore, these results indicate that the petiole of the radical leaves in the riverside population has a fixed trait of shorter length, despite having a similar diameter at the base.

**FIGURE 4:**
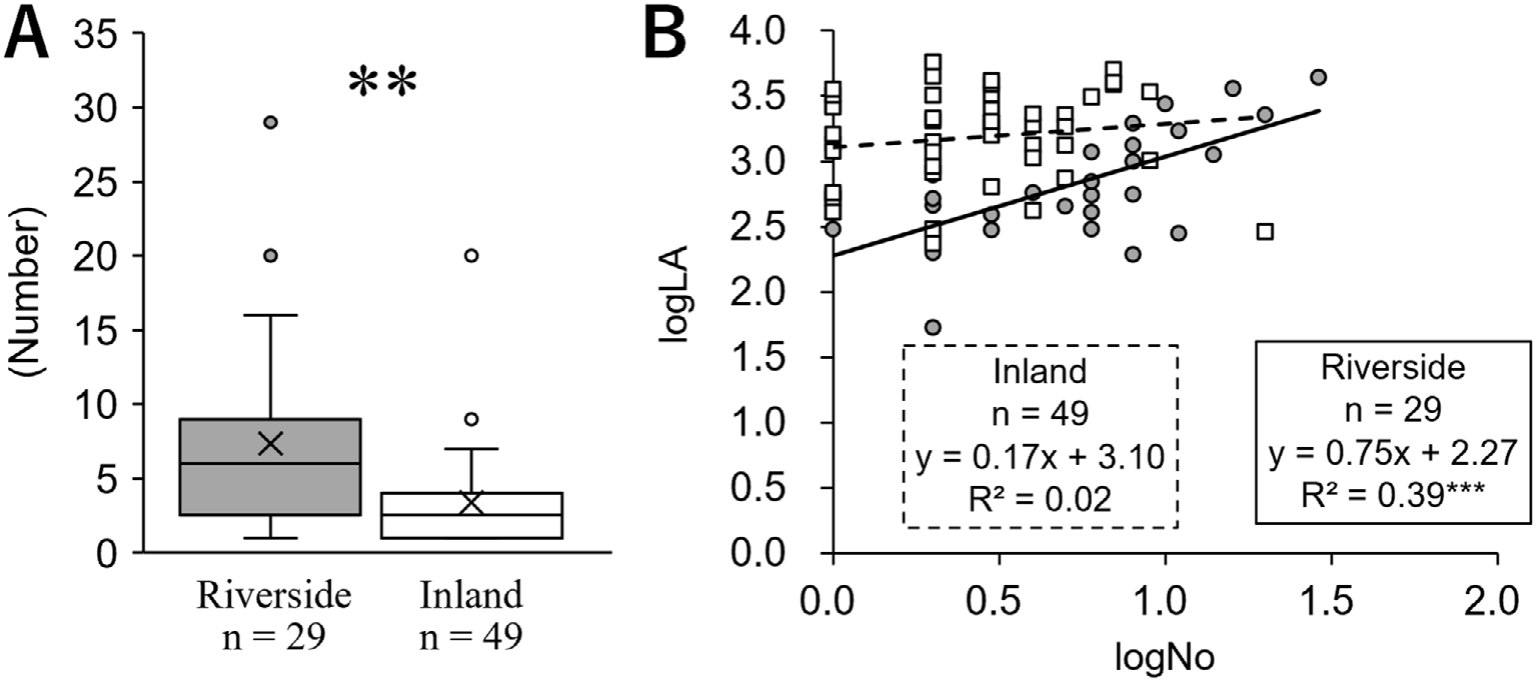
Comparison of number of radical leaves. (A) number of radical leaves. (B) Correlation between number (logNo) and total leaf area (logLA) of radical leaves. The riverside population is indicated by grey circles and solid lines, while the inland population is indicated by white squares and dashed lines. The statistical significance of comparisons between two groups and the relationship between two variables is as follows: **, *p* < 0.01, n.s., not significant.

The number of radical leaves per individual in the riverside population was 7.34 ± 1.14, whereas that in the inland population was significantly lower at 3.38 ± 0.44 (*p* < 0.01; Figure 4A). The relationship between the total leaf area and the number of leaves per individual in the radical leaves showed a statistically significant positive correlation in the riverside population (riverside: *p* < 0.001, inland: *p* = 0.29). Additionally, the results of the ANCOVA revealed a significant interaction effect (*p* < 0.05). This suggests that, unlike the inland population, where total leaf area increases with the size of individual leaves, the radical leaves of the riverside population increase total leaf area by increasing the number of leaves (Figure 4B).

### 3.2 Morphological Measurements of the Cauline Leaves and Stem

**FIGURE 5:**
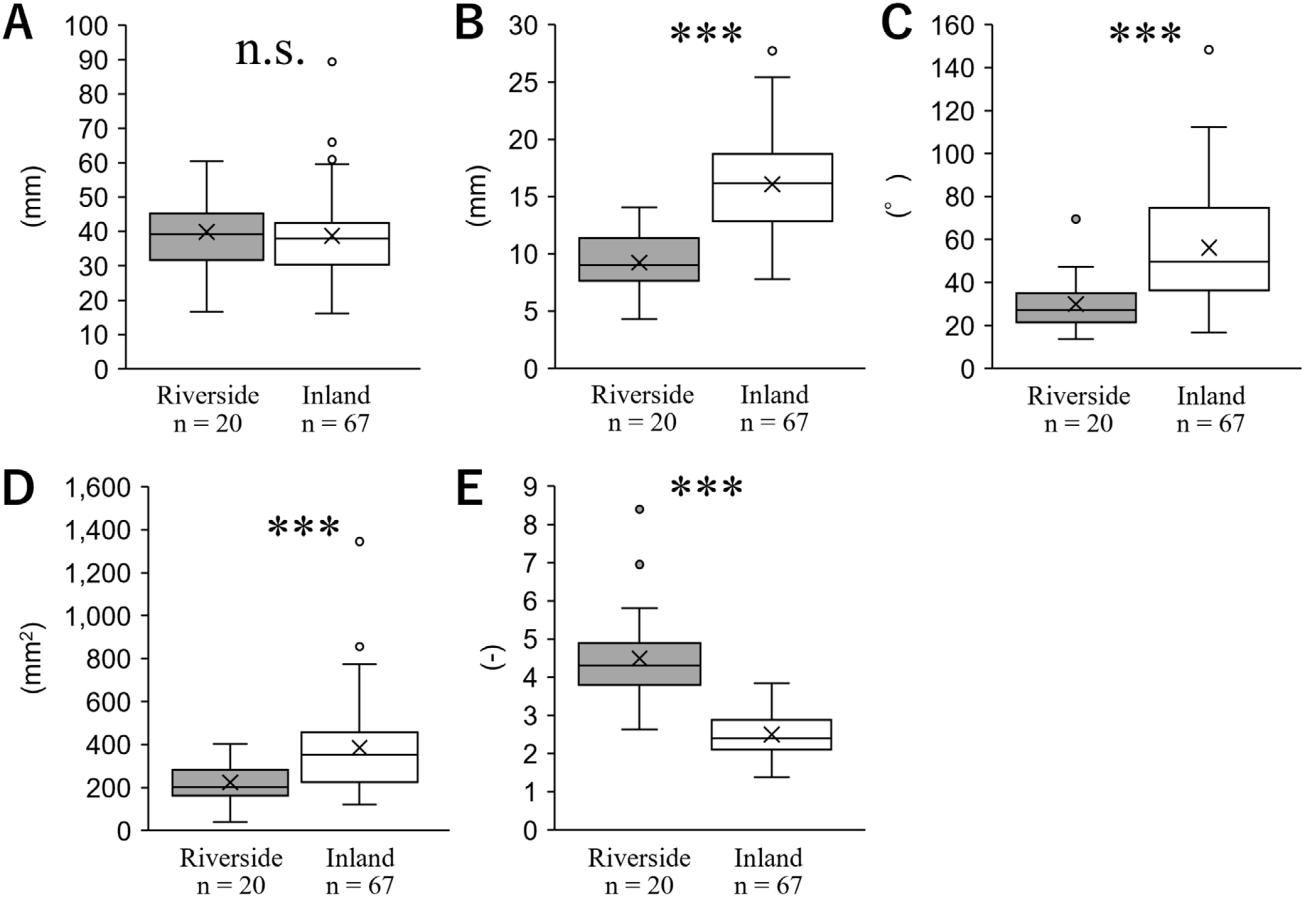
Comparisons of cauline leaves in cultivation. (A) length, (B) width, (C) angle at lamina base, (D) leaf area, (E) leaf index. The statistical significance is as follows: ***, *p* < 0.001, n.s., not significant.

No significant difference in leaf length was observed between the two populations (*p* = 0.70), with the riverside population measuring 39.88 ± 2.38 and the inland population measuring 38.73 ± 1.49 (Figure 5A). The leaf width of the riverside population was 9.24 ± 0.58, whereas that of the inland population was significantly wider at 16.06 ± 0.52 (*p* < 0.001; Figure 5B). The angle at the lamina base in the riverside population was 29.94 ± 2.91, while the inland population had a significantly wider angle at 56.14 ± 3.09 (*p* < 0.001; Figure 5C). The leaf area of the riverside population was 224.12 ± 21.56, whereas that of the inland population was significantly larger at 385.17 ± 25.21 (*p* < 0.001; Figure 5D). The leaf index of the riverside population was 4.48 ± 0.29, whereas that of the inland population was significantly lower at 2.50 ± 0.07 (*p* < 0.001; Figure 5E). These results suggest that, although no significant differences were observed in cauline leaves, the cultivated riverside population's cauline leaves have a fixed trait of narrower leaf width and a more elongated shape (Figure 5).

**FIGURE 6:**
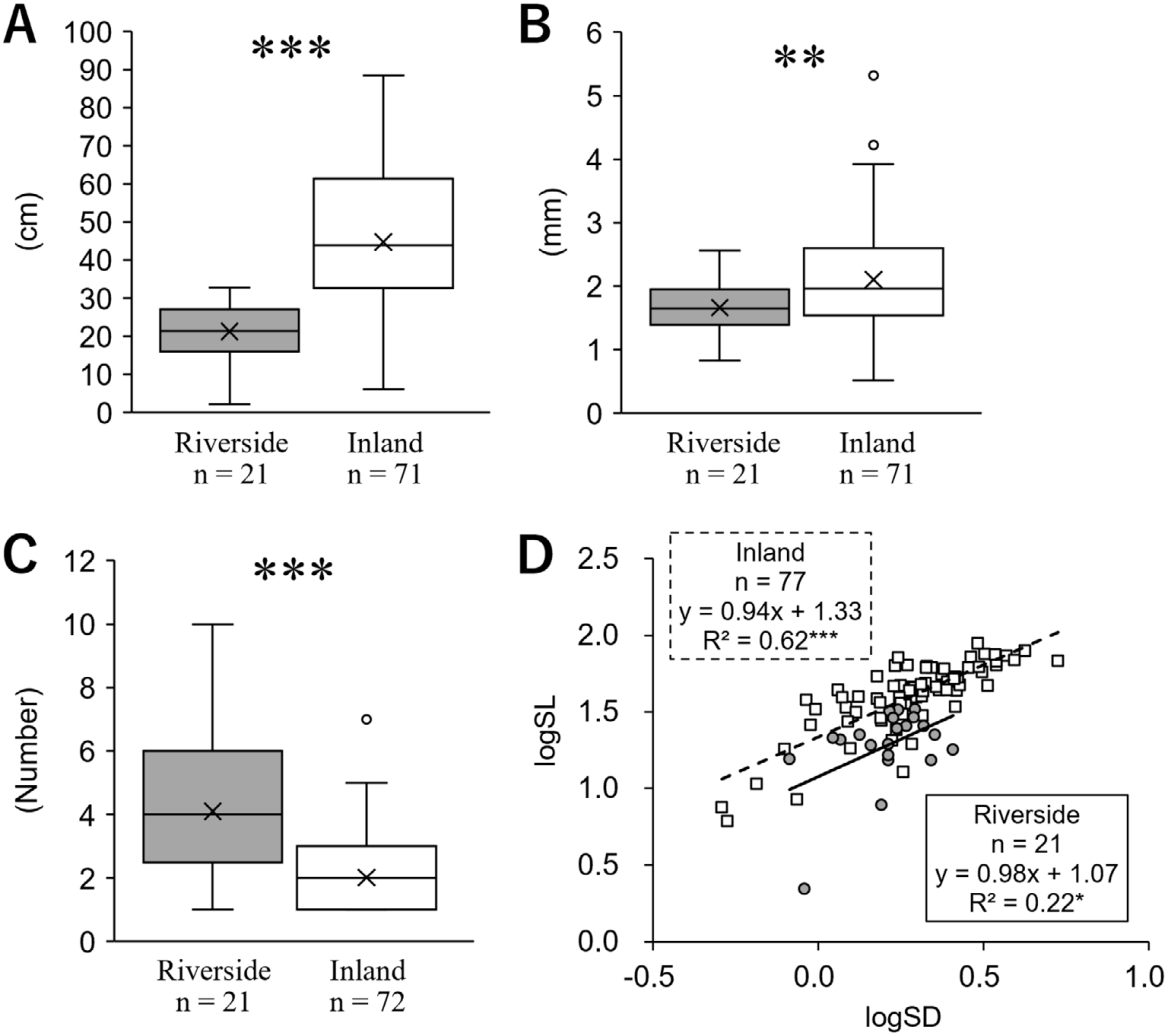
Comparisons of stems. (A) length, (B) basal diameter. (C) number of rosette‐leaf branches. (D) Correlation between basal diameter (logSD) and length (logSL) of stem. The statistical significance of comparisons between two groups and the relationship between two variables is as follows: *p < 0.05; **p < 0.01, ***p < 0.001, n.s., not significant.

The stem length of the riverside population was 21.24 ± 1.71, whereas that of the inland population was significantly longer at 44.77 ± 2.20 (*p* < 0.001; Figure 6A). The basal diameter of the stem in the riverside population was 1.66 ± 0.09, while that of the inland population was significantly thicker at 2.10 ± 0.01 (*p* < 0.01; Figure 6B). The number of rosette‐leaf branches in the riverside population was 4.09 ± 0.51, whereas that in the inland population was significantly lower at 2.01 ± 0.15 (*p* < 0.001; Figure 6C). The relationship between stem length and basal diameter of the stem showed a statistically significant positive correlation in both populations (riverside: *p* < 0.05, inland: *p* < 0.001). Additionally, the results of the ANCOVA revealed a significant difference in the intercept (*p* < 0.001), but no interaction effect was observed (*p* = 0.89). This suggests that the slope of the relationship between stem length and diameter at the base of the stem is the same regardless of the growing environment, while the intercept of stem length differs depending on the environment (Figure 6D). Furthermore, these results suggest that the stem length of the riverside population is a genetically fixed trait, as the stems are significantly shorter than those of the inland population when the basal diameter is comparable. In addition, when stem length is held constant, the basal diameter of the riverside population tends to be significantly greater.

### 3.3 Comparison of Cultivated and Wild Populations of the Cauline Leaves

**FIGURE 7:**
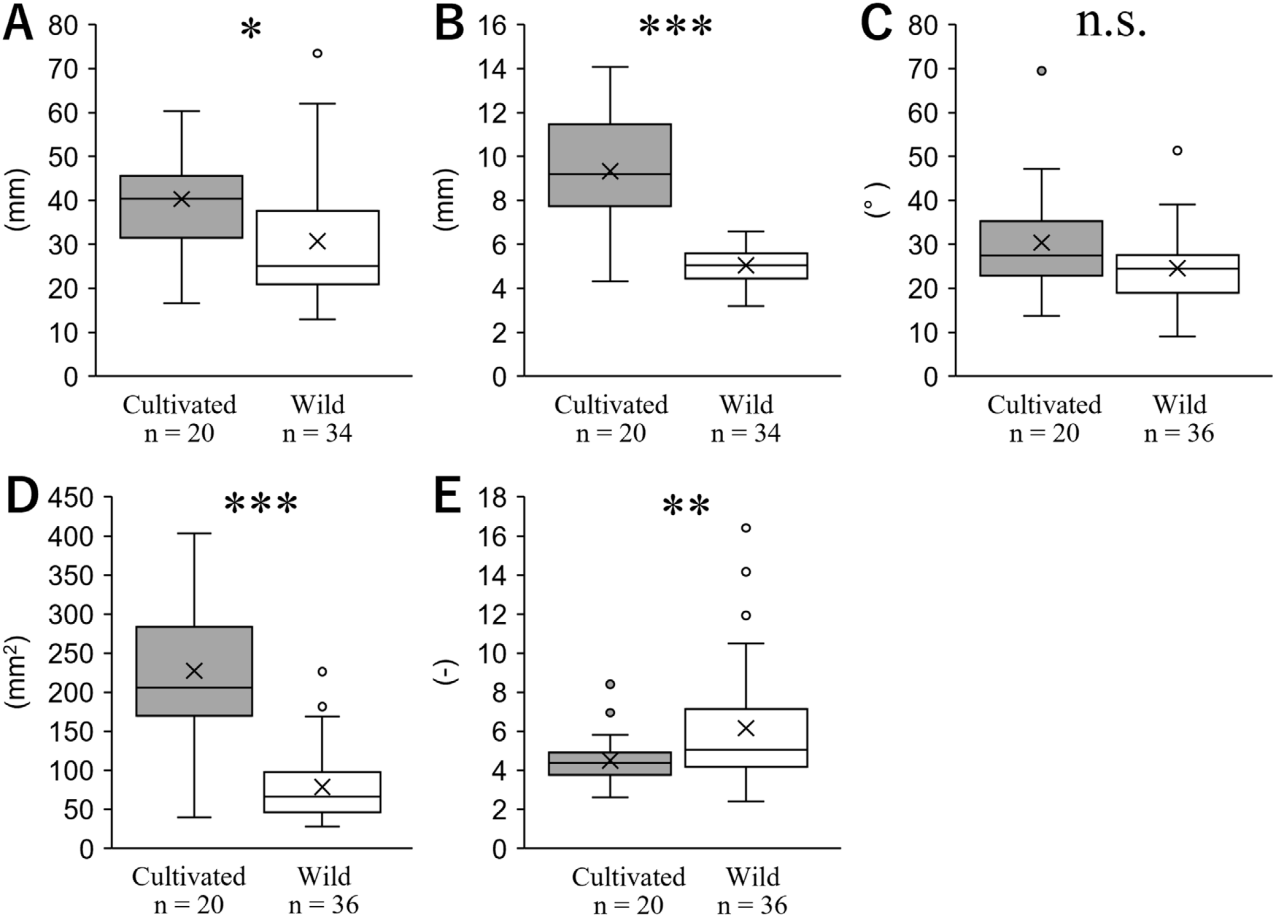
Comparisons of cauline leaves in riverside populations between cultivated and wild populations. (A) length, (B) width, (C) angle at lamina base, (D) leaf area, (E) leaf index. The statistical significance is as follows: *p < 0.05; **p < 0.01, ***p < 0.001, n.s., not significant.

The morphological characteristics of the cauline leaves in the riverside population were compared between the cultivated and wild populations, as shown in Figure 7. The leaf length of the cultivated population was 39.88 ± 2.38, whereas that of the wild population was significantly shorter at 30.08 ± 2.50 (*p* < 0.05; Figure 7A). The leaf width of the cultivated population was 9.24 ± 0.58, whereas that of the wild population was significantly narrower at 5.12 ± 0.13 (*p* < 0.001; Figure 7B). No significant difference in the angle at the lamina base was observed between the two populations (*p* = 0.10), with the cultivated population having an angle of 29.94 ± 2.91 and the wild population 24.60 ± 1.37 (Figure 7C). The leaf area of the cultivated population was 224.12 ± 21.56, whereas that of the wild population was significantly smaller at 78.78 ± 7.49 (*p* < 0.001; Figure 7D). The leaf index of the cultivated population was 4.48 ± 0.29, whereas that of the wild population was significantly higher at 6.15 ± 0.51 (*p* < 0.001; Figure 7E). These results suggest that, while the angle at the lamina base forms a similar angle, the leaf size and shape of the wild population are smaller and more elongated than those of the cultivated population.

**FIGURE 8:**
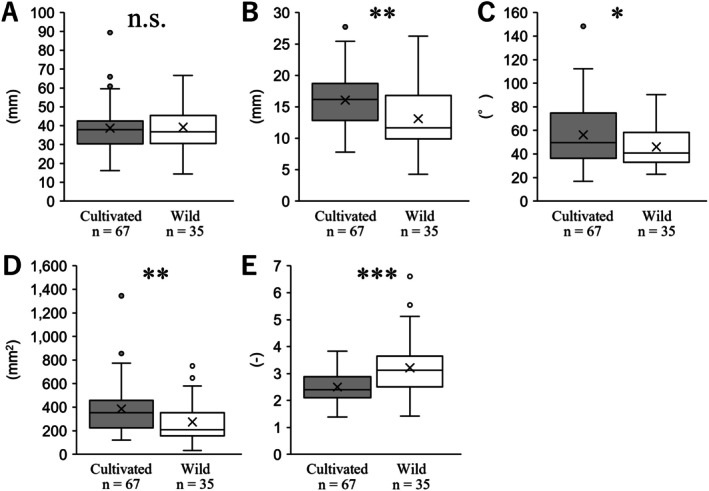
Comparisons of cauline leaves in inland populations between cultivated and wild populations. (A) length, (B) width, (C) angle at lamina base, (D) leaf area, (E) leaf index. The statistical significance is as follows: *p < 0.05; **p < 0.01, ***p < 0.001, n.s., not significant.

The morphological characteristics of the cauline leaves in the inland population were compared between the cultivated and wild populations, as shown in Figure 8. No significant difference in leaf area was observed between the two populations (*p* = 0.85), with the cultivated population measuring 38.73 ± 1.49 and the wild population measuring 39.22 ± 2.22 (Figure 8A). The leaf width of the cultivated population was 16.06 ± 0.52, whereas that of the wild population was significantly narrower at 13.09 ± 0.86 (*p* < 0.01; Figure 8B). The angle at the lamina base of the cultivated population was 56.14 ± 3.09, while that of the wild population was significantly narrower at 46.07 ± 2.96 (*p* < 0.05; Figure 8C). The leaf area of the cultivated population was 385.17 ± 25.21, whereas that of the wild population was significantly smaller at 275.41 ± 29.89 (*p* < 0.01; Figure 8D). The leaf index of the cultivated population was 2.50 ± 0.07, whereas that of the wild population was significantly higher at 3.21 ± 0.18 (*p* < 0.001; Figure 8E). These results suggest that, although the leaf length in the inland population forms a similar length, the leaf size and shape of the wild population are smaller and more elongated compared to the cultivated population.

## 4 Discussion

### 4.1 Comparison of Radical Leaves Between the Riverside and Control Populations

Our results indicated that the lamina of radical leaves in the riverside population of *Ad. triphylla* var. *japonica* was narrower and smaller, with an angle to the base of the lamina (Figure 2). These traits are similar to those reported for cauline leaves (Ohga, Muroi, Hayakawa, Ito, et al. 2012), indicating that the radical leaves of riverside populations exhibit a morphology that reduces water flow stress during river floods. In addition, the petioles of the radical leaves in the riverside population of *Ad. triphylla* var. *japonica* were significantly shorter than those in the control population (Figure 3A), and at the same petiole length, the petiole bases were thicker (Figure 3C). The question arises whether morphological change in the petioles of the radical leaves of this riverside population leads to reduced water flow stress, as in the lamina. For radical leaves, Anten et al. (2010) compared morphological and mechanical traits under wind stress using *Plantago major* L. (Plantaginaceae) and showed that the petiole length was shorter, but the petiole diameter was not different from that of the controls. Shiba et al. (2023) reported that radical leaves of *F. japonicum* var. *japonicum* (Asteraceae) exhibited reduced lamina size and shortened petioles while maintaining their diameter with the increase in wind speed. Thus, our results showed many similarities to the morphological changes under wind stress, such as the reduction in lamina area and the shortening of petioles of radical leaves, but the riverside population of *Ad. triphylla* var. *japonica* with thick petioles differed from each other. Petioles can serve multiple functions, such as providing mechanical support for the lamina (Yamada et al. 1999) and adjusting leaf angle or leaf orientation to adapt to the variation in environments (Falster and Westoby 2003). Petiole lodging of radical leaves can lead to the physical collapse of the photosynthetic capacity and can occur spontaneously through mechanical instability of the leaf structure by external forces. Wind and water flow stress are the primary environmental factors responsible for petiole lodging, which occurs when plants are subjected to forces greater than the maximum force that the petiole can withstand before breaking. Some studies have shown an avoidance strategy against mechanical stress by reducing the thickness and bending rigidity of the stems and petioles (Jaffe et al. 1984; Cordero 1999; Liu et al. 2007; Paul‐Victor and Rowe 2011), and some plant species show resistance to mechanical stress by increasing the stem and petiole cross‐sectional area and tissue strength (Biddington 1986; Smith and Ennos 2003; Anten et al. 2010). This suggests that the petioles of radical leaves in the riverside population of *Ad. triphylla* var. *japonica* were less likely to bend as the thickness increased. Therefore, our results show that the riverside population of *Ad. triphylla* var. *japonica* acquired resistance to water flow stress along rivers, indicating that the adaptation processes along rivers were different from previous rheophytic results with petiole flexibility of *O. lancea* (Shiba and Fukuda 2024).

Our results indicated that the number of radical leaves in the riverside population of *Ad. triphylla* var. *japonica* was significantly higher than that in the inland population (Figure 4A). In addition, the decrease in the lamina area of radical leaves in the riverside population was compensated for by an increase in the number of radical leaves; therefore, the total lamina area in the riverside population was closer to that of the inland areas (Figure 4B). In particular, the increase in the number of radical leaves in the inland population had almost no effect on the increase in total lamina area (Figure 4B), revealing that radical leaves in the riverside population have a different strategy from those in the inland population. Petiole growth is one of the most obvious changes to diminish self‐shading at the expense of support because it can send the lamina to a higher position and adjust the angle of the lamina on a branch to avoid overlapping with its neighbors (King and Maindonald 1999; Bell and Galloway 2007; Sarlikioti et al. 2011; Zhong et al. 2019). This was considered to reduce the risk of overlapping laminae in the inland population of the *Ad. triphylla* var. *japonica* because of its longer petioles and small number of radical leaves. The leaves of the riverside populations had a narrow lamina of radical leaves, but an increased number of leaves increased the risk of lamina overlap. Why did the riverside population increase the number of radical leaves so much, given this risk? One answer is that the riverside population has several opportunities to lose radical leaves due to strong water flow stress caused by rising water levels; therefore, the riverside population of *Ad. triphylla* var. *japonica* may produce several radical leaves, leaving some alone, even when affected by high water levels. At first glance, this strategy seems wasteful in terms of photosynthetic efficiency, but it may have become established because few competitors in the areas along the river are subject to water flow stress.

### 4.2 Comparison of Cauline Leaves and Stems Between the Riverside and Control Populations

Plants have developed various adaptations to cope with strong stress exposure; in particular, reducing stress on the stem plays an important role in maintaining reproductive organs, such as flowers and inflorescences (Shiba, Kobayashi, et al. 2024). For example, in the case of wind stress, plants adapt by shortening stems and petioles and making leaves smaller to reduce stress, which is often achieved through phenotypic plasticity (Nagashima and Hikosaka 2011; Shiba et al. 2023; Shiba, Arihara, et al. 2024; Shiba, Kobayashi, et al. 2024). This indicates that reducing stress on each cauline leaf could significantly reduce stress on the stem. The question then arises whether plants adapt to the stress caused by water flow through phenotypic plasticity. Ohga, Muroi, Hayakawa, et al. (2012) reported that riverside populations of *Ad. triphylla* var. *japonica* have narrow leaves compared to its cauline leaves. Our result of cultivating seeds collected from this population was that the riverside population had significantly narrower cauline leaves than the inland population (Figure 5), revealing that the differences in leaf morphology observed in the field were genetically fixed rather than caused by plasticity. However, our comparison between the cultivated and wild riverside populations showed that the former had significantly longer and wider cauline leaves (Figure 7). Considering leaves are very flexible, unlike branches and stems, some environmental factors have been shown to affect various cauline leaf traits under field conditions, among which wind particularly affects cauline leaf number and size (Niklas 1996; Anten et al. 2010; Shiba et al. 2023; Shiba, Arihara, et al. 2024; Shiba, Kobayashi, et al. 2024). Therefore, the length, width, and angle of the lamina base of the cultivated and wild species were significantly different, as reflected in the leaf shape index (Figures 7 and 8). Tsukaya (2002) proposed a leaf index value calculated as the ratio of leaf length to leaf width, which has been used in various studies to compare leaf silhouettes (Yamada et al. 2011; Ohga, Muroi, Hayakawa, Ito, et al. 2012; Ueda et al. 2012; Yokoyama et al. 2012; Kumekawa et al. 2013; Matsui et al. 2013; Shiba et al. 2021, 2022a, 2022b, 2022c). Although the leaf shape indices in these studies differed greatly between the comparison environments, the differences between the cultivated and wild populations in this study were very small (Figures 7 and 8), indicating that the riverside population of *Ad. triphylla* var. *japonica* was formed by individuals with genetically narrower leaf widths, and the leaf width did not reach the same level as the inland population, based on the comparisons of cultivated and wild populations. In rheophytic woody plants, *R. ripense* has markedly narrower leaves than its closely related inland species *R. macrosepalum*, and this stenophyllization has been shown to result from a reduction in cell number rather than a decrease in cell size (Ueda et al. 2012). In addition, *R. ripense* exhibits increased leaf thickness and stomatal density, which have been suggested as morphological and anatomical adaptations not only to water flow stress but also to the high‐light environments along rivers. In contrast, Nomura et al. (2006) reported that in the rheophytic herbaceous plant *F. japonicum* var. *luchuense*, stenophylly enhances leaf mechanical toughness but at the same time reduces photosynthetic efficiency per unit dry mass, suggesting that stenophyllous traits may function in response to both light conditions and water flow stress in riparian habitats. In the riverside population of *Ad. triphylla* var. *japonica* examined in this study, it remains unclear whether stenophylly is attributable to a genetically fixed reduction in cell number. Nevertheless, future cellular‐level analyses will be required to evaluate this possibility, particularly from the perspective of a potential trade‐off between photosynthetic efficiency and stress tolerance.

Our results reveal that the stems of riverside populations of *Ad. triphylla* var. *japonica* were shorter than those of the inland population (Figure 6), and at the same stem length, the stem bases were thicker (Figure 6D), suggesting a strategy to make the stems of the former stronger and more resistant to water flow stress rather than making them more flexible and reduced, as with the aforementioned radical leaves. A significant difference was also observed between the riverside and inland populations in the number of rosette leaf branches from the rhizome, with the former having significantly more rosette leaf branches (Figure 6C). The rosette‐leaf branch is an important indicator of fitness because it affects individual yield (Wang and Li 2011), and the differences in rosette‐leaf branches between populations seem to have several similarities to those in the petioles of radical leaves (Figures 3, 4 and 6). This indicates that they tried to minimize the moment acting on each by not growing as high above the ground as possible. These results suggest that it is more reasonable to consider that the aboveground parts of *Ad. triphylla* var. *japonica* along rivers have changed to tolerate the water flow stress caused by flooding with sturdy stems, rosette‐leaf branches, and petioles, rather than reducing it with flexibility. However, it is unclear what genetic background exists for the formation of such rosette‐leaf branches in the riverside population of *Ad. triphylla* var. *japonica*. In the model plant *Arabidopsis thaliana* (L.) Heynh., Aguilar‐Martínez et al. (2007) showed that the *brc1* mutant had a significantly higher number of rosette branches compared to wild‐type plants, and that the double mutant *brc1‐2 brc2‐1* exhibited a phenotype similar to that of the strong *brc1* mutant. The increase in rosette branches was due to an increase in the frequency of bud elongation and not an increase in the number of vegetative nodes, indicating that these genes inhibit the elongation of rosette branches (Aguilar‐Martínez et al. 2007). The TCP gene family, which includes these genes, has a conserved basic helix–loop–helix (bHLH) structure related to DNA binding function (Cubas et al. 1999). For example, Luo et al. (1996) reported that the *cyc* (*cycloidea*) gene of *Antirrhinum majus* L. (Scrophulariaceae), which belongs to the TCP gene family, is expressed very early in the dorsal region of the floral meristem and controls floral asymmetry by influencing the growth rate and initiation of primordia. Moreover, Doebley et al. (1997) showed that branched *tb1* (*teosinte branched1*) suppresses the growth of axillary organs and that this change in gene regulation underlies the evolutionary divergence of maize ( *Zea mays* L. subsp. *mays*) and teosinte ( *Z. mays* subsp. *mexicana* (Schrad.) H. H. Iltis) (Poaceae), indicating that the TCP gene family is involved in the growth of organs and tissues in phylogenetically different plant groups. This gene family exists in early land plants, and its function in controlling growth and development is conserved in plant species ranging from mosses to angiosperms (Wang et al. 2022), and several studies have been conducted in numerous plant species, including the moss *Physcomitrella patens* (Hedw.) Bruch et Schimp (Ortiz‐Ramírez et al. 2016), the fern *Selaginella mollendorffii* Hieron. (Navaud et al. 2007), gymnosperm *Ginkgo biloba* L. (Yu et al. 2022), monocotyledonous angiosperm *Oryza sativa* L. (Kosugi and Ohashi 1997), and *Phyllostachys edulis* (Carrière) Houz. (Liu et al. 2018), as well as the dicotyledonous plant *Liliodendron chinense* (Hemsl.) Sarg. (Magnoliaceae) (Hwarari et al. 2022), *Ar. thaliana* (Brassicaceae) (Aggarwal et al. 2010), *Peralia lobata* (Willd.) Ohwi and *Wisteria sinensis* (Sims) DC. (Fabaceae) (Fukuda et al. 2003), *Solanum lycopersicum* L. (Solanaceae) (Parapunova et al. 2014), and *Taraxacum officinale* Weber ex F. H. Wigg (Asteraceae) (Xiong et al. 2023). However, in some plant groups, they have not yet been identified (Manassero et al. 2013). Therefore, it was expected that this TCP gene family would also be involved in the morphological differences between the wild type (inland population) and the mutant type (riverside population) of *Ad. triphylla* var. *japonica*, and a comparison of the gene levels between the wild‐type (inland population) and such analyses would be the basis for evolutionary developmental (Evo‐Devo) or ecological developmental (Eco‐Devo) research. They can be considered a mode of adaptive evolution of *the Ad. triphylla* var. *japonica* into the riverside because they are brought about by genetic changes based on comparisons of cultivated and wild populations.

## 5 Summary and Further Perspective

Our study revealed that the riverside populations of *Ad. triphylla* var. *japonica* had adapted through several genetic and morphological changes. This is the first report to show that this adaptation mode may be resistant to water flow stress during river floods. In future studies, it will be important to conduct open‐channel flow experiments to quantitatively evaluate the extent to which stenophyllization reduces hydrodynamic drag under running water conditions. Such experimental verification would help clarify how the stenophyllous traits reported in riparian plants function not merely as morphological characteristics but as functional morphologies that contribute to the reduction of water flow stress. Moreover, our common garden experiment strongly suggests genetic fixation of leaf morphology. Nevertheless, reciprocal transplant experiments will be useful in future studies to assess potential effects of local soil and microbial communities. In particular, while wind stress can often be mitigated through phenotypic plasticity, water flow stress cannot be overcome by plasticity alone and requires genetic fixation; therefore, future studies should also examine why water flow stress acts as a much stronger selective force than wind stress. In addition to these morphological aspects, Shiuchi (2019) showed that seeds of the riverside population of *Ad. triphylla* var. *japonica* had germinated without dormancy even at high temperatures of over 20°C, while the seeds of terrestrial plants showed dormancy, suggesting that early germination characteristics of the riverside population had an advantage in adapting to the unstable water environment along rivers where water levels and flow increase occur occasionally. Considering these results, *Ad. triphylla* var. *japonica* that have been genetically fixed to adapt to rivers cannot grow outside areas subject to water flow stress, and their distribution is limited. Such populations have only been reported in a few places in Japan (Ohga, Muroi, Hayakawa, Ito, et al. 2012; Shiuchi 2019). There is, therefore, a risk of extinction if the water volume of rivers is regulated by the construction of dams or other structures upstream of their habitats, indicating that it is also necessary for future research from a conservation perspective.

The present study focused on one of the riverside populations of *Ad. triphylla* that had previously been reported as a rheophytic ecotype by Ohga, Muroi, Hayakawa, Ito, et al. (2012). In fact, that study documented riverside populations from several other localities in Shikoku, Japan, suggesting that leaf morphology is likely to be genetically fixed in those populations as well. Furthermore, Shiuchi (2019) also reported the presence of a rheophytic ecotype in Honshu, Japan, indicating that this ecotype is more widely distributed. Therefore, future studies should include additional populations and, ideally, incorporate genetic markers such as SNP analyses to more rigorously evaluate the extent of genetic differentiation underlying morphological adaptation. *Ad. triphylla* var. *japonica* has been reported to adapt by growing narrower leaves in serpentine soils with high concentrations of potentially toxic Ni and Mg, low concentrations of plant nutrients, and low Ca: Mg ratios (Ohga, Muroi, Hayakawa, Yokoyama, et al. 2012), and by growing thicker leaves in coastal areas exposed to different levels of salt spray and high winds (Ohga et al. 2013). In addition, this species is described as an alpine‐type, *Ad. triphylla* var. *japonica* f. *albiflora* (Tatew.) H. hara is an interesting subject for considering its adaptation to alpine environments (Okazaki 1993). Investigating whether the morphological changes that enable adaptation to such a variety of environments exhibit phenotypic plasticity may support future research on Evo‐Devo and/or Eco‐Devo.

## Author Contributions

**Iori Yajima:** conceptualization (equal), formal analysis (equal), investigation (equal), methodology (supporting), writing – review and editing (equal). **Masayuki Shiba:** conceptualization (equal), formal analysis (equal), funding acquisition (equal), investigation (lead), methodology (lead), project administration (lead), software (lead), supervision (lead), writing – original draft (supporting), writing – review and editing (equal). **Kyohei Ohga:** investigation (supporting), resources (equal), writing – review and editing (equal). **Yoshimasa Kumekawa:** investigation (supporting), resources (equal), writing – review and editing (equal). **Tatsuya Fukuda:** conceptualization (equal), funding acquisition (equal), supervision (supporting), writing – original draft (lead).

## Conflicts of Interest

The authors declare no conflicts of interest.

## Supporting information


**Data S1:** ece372222‐sup‐0001‐DataS1.xlsx.

## Data Availability

All the required data is uploaded as supporting information—S1.
